# Pathophysiological Role of Transient Receptor Potential Ankyrin 1 in a Mouse Long-Lasting Cystitis Model Induced by an Intravesical Injection of Hydrogen Peroxide

**DOI:** 10.3389/fphys.2017.00877

**Published:** 2017-11-07

**Authors:** Shohei Oyama, Koji Dogishi, Mizuki Kodera, Masashi Kakae, Kazuki Nagayasu, Hisashi Shirakawa, Takayuki Nakagawa, Shuji Kaneko

**Affiliations:** ^1^Department of Molecular Pharmacology, Graduate School of Pharmaceutical Sciences, Kyoto University, Kyoto, Japan; ^2^Department of Clinical Pharmacology and Therapeutics, Kyoto University Hospital, Kyoto, Japan

**Keywords:** TRPA1, cystitis, hydrogen peroxide, mouse model, chronic inflammatory bladder, gene knockout

## Abstract

Chronic inflammatory bladder disorders, such as interstitial cystitis/bladder pain syndrome, are associated with poor quality of life. The exact pathological processes remain unclear, but accumulating evidence suggests that reactive oxidative species (ROS) are involved in urinary bladder disorders. Transient receptor potential ankyrin 1 (TRPA1), the most sensitive TRP channel to ROS, was shown to be responsible for urinary bladder abnormalities and hyperalgesia in an acute cystitis model. However, the roles of TRPA1 in chronic inflammatory bladder are not fully understood. We previously established a novel mouse cystitis model induced by intravesical injection of hydrogen peroxide (H_2_O_2_), resulting in long-lasting frequent urination, bladder inflammation, pain-related behavior, and histopathological changes. In the present study, we investigated the pathophysiological role of TRPA1 in the H_2_O_2_-induced long-lasting cystitis mouse model. Under anesthesia, 1.5% H_2_O_2_ solution was introduced transurethrally into the bladder of female wild-type (WT) and TRPA1-knockout mice and maintained for 30 min. This increased the number of voids in WT mice at 1 and 7 days after injection, but reduced the number in TRPA1-knockout mice at 1 day but not 7 days after injection. Spontaneous locomotor activities (increase in freezing time and decrease in distance moved) were reduced at 3 h after injection in WT mice, whereas the spontaneous visceral pain-related behaviors were attenuated in TRPA1-knockout mice. Furthermore, upregulation of *c-fos* mRNA in the spinal cord at 1 day after injection was observed in WT but not TRPA1-knockout mice. However, there was no difference in histopathological changes in the urinary bladder, such as edematous thickening in the submucosa, between WT and TRPA1-knockout mice at 1 or 7 days after injection. Finally, *Trpa1* mRNA levels in the L5-S1 dorsal root ganglion were not altered, but levels in the urinary bladder were drastically increased at 1 and 7 days after injection. Taken together, these results suggest that TRPA1 contributes to acute bladder hyperactivity such as frequent urination and bladder pain, but does not appear to play a major role in the pathological processes of long-lasting cystitis.

## Introduction

Lower urinary tract symptoms, such as urinary frequency, urgency, nocturia, and abdominal visceral pain, lead to impaired quality of life. These symptoms are features of chronic inflammatory bladder disorders including interstitial cystitis/bladder pain syndrome (IC/BPS). Many hypotheses about the pathogenesis of chronic cystitis have been proposed, such as urothelial dysfunction, inflammation, neural hyperactivity, an autoimmune response, and toxic urinary agents (Homma et al., 2016). However, the exact pathological processes involved remain unclear.

Accumulating evidence suggests that reactive oxidative species (ROS) contribute to bladder disorders. Excess ROS are a feature of various bladder pathological conditions including bladder outlet obstruction (Lin et al., 2005), bladder ischemia/reperfusion (Yu et al., 2004), and bladder inflammation models (Chien et al., 2003). Furthermore, ROS are abundantly produced by inflammatory cells such as macrophages, neutrophils, and mast cells when they infiltrate an inflamed bladder (Brooks et al., 1999; Winterbourn, 2002; Chien et al., 2003; Ndengele et al., 2005). ROS induce bladder hyperactivity by activating capsaicin-sensitive C-fiber afferent pathways (Masuda et al., 2008; Nicholas et al., 2017). In cyclophosphamide- or ifosfamide-induced acute cystitis animal models, the metabolite acrolein enters the urothelium and causes bladder inflammation, which is prevented by ROS scavengers or antioxidants (Yildirim et al., 2004; Batista et al., 2007; Song et al., 2014). In addition, a human study demonstrate that the serum total antioxidant capacity in IC/BPS patients is lower than that in healthy controls (Ener et al., 2015). Thus, it is likely that ROS play a critical role in the etiology and/or pathology of chronic cystitis.

Transient receptor potential ankyrin 1 (TRPA1), a non-selective cation channel, is highly expressed in a subset of nociceptive C-fibers where it acts as a polymodal nociceptor (Wu et al., 2010). TRPA1 is activated by various irritants and oxidative stimuli including ROS, and contributes to nociceptive and inflammatory pain generation (Jordt et al., 2004; Bautista et al., 2006; Andersson et al., 2008; Sawada et al., 2008). In the lower urinary tract, TRPA1 is expressed in the urothelium or detrusor of the urinary bladder in addition to the C-fibers (Du et al., 2008; Streng et al., 2008). This is because intravenous administration of a TRPA1 antagonist does not alter the voiding function, while intravesical infusion of a TRPA1 agonist increases the micturition frequency (Streng et al., 2008; Minagawa et al., 2014), indicating that TRPA1 does not play a major role in bladder function under physiological conditions. By contrast, in a cyclophosphamide-induced cystitis model, bladder hyperalgesia, and voiding frequency are caused by activation of TRPA1 (Meotti et al., 2013; DeBerry et al., 2014). Moreover, human studies reveal that *Trpa1* mRNA levels in the urinary bladder are markedly elevated in patients with IC/BPS (Homma et al., 2013) and bladder outlet obstruction (Du et al., 2008). Thus, it is likely that ROS-sensitive TRPA1 may play a key role in the pathogenesis or pathology of chronic cystitis, although this is not fully understood at present.

We previously established a novel long-lasting cystitis mouse model by intravesical injection of hydrogen peroxide (H_2_O_2_) (Homan et al., 2013). The H_2_O_2_-induced long-lasting cystitis model is characterized by long-lasting frequent urination, bladder inflammation, pain-related behavior, and histopathological changes (Homan et al., 2013; Dogishi et al., 2015). In the present study, we investigated the pathophysiological roles of TRPA1 in the H_2_O_2_-induced long-lasting cystitis model using TRPA1-knockout (KO) mice.

## Materials and methods

### Animals

All experiments were performed according to the ethical guidelines recommended by the Kyoto University Animal Research Committee. The protocol was approved by the Kyoto University Animal Research Committee (permit number: 2015–40, 2016–40). *Trpa1*^+/+^ (wild-type; WT) and *Trpa1*^−/−^ (TRPA1-KO) mice lines were bred from heterozygous mice with a C57BL/6 × 129 S1 background that were obtained from Jackson Laboratory (Bar Harbor, ME). Mouse lines were backcrossed to C57BL/6 J mice for at least 10 generations, and genotyped by genomic PCR using primers 5′-tcatctgggcaacaatgtcacctgct-3′ and 5′-tcctgcaagggtgattgcgttgtcta-3′. Female WT and TRPA1-KO mice aged between 5 and 6 weeks old were used, while female C57BL/6 J mice of the same age were purchased from Japan SLC (Shizuoka, Japan) and used in some experiments. All mice were housed under constant ambient temperature (24 ± 1°C) and humidity (55 ± 20%), with an alternating 12 h light/dark cycle (lights came on automatically at 8:00 a.m.). Food and water were freely available.

### H_2_O_2_-induced cystitis model

The H_2_O_2_-induced cystitis model was generated as previously reported (Homan et al., 2013). Briefly, under 2–3% isoflurane (Pfizer, NY) anesthesia, a polyethylene tube (PE-10; Clay-Adams, Parsippany, NJ) was introduced into the bladder transurethrally and the lower abdomen was pressed gently to withdraw urine. Next, 50 μL of 1.5% H_2_O_2_ solution (Wako Pure Chemical Industries, Osaka, Japan) in sterile saline was introduced into the bladder through the catheter. The H_2_O_2_ solution was drained from the bladder after 30 min by pressing the lower abdomen.

### Measurement of the number of voids and spontaneous locomotor activities

Mice were kept in an individual plastic cage (10 × 20 × 30 cm: width × length × height) lined with filter paper (Advantec Chromatography Paper No. 50; Toyo Roshi Kaisha, Ltd., Tokyo, Japan) and allowed to acclimate for 30 min before experiments. After replacing the filter paper, the mouse was videotaped for 15 min, and the number of voids was quantified from the videotape by counting urine spots on the filter paper. Subsequently, freezing time and move distance were analyzed using the ANY-maze video tracking system (Stoelting Co., Wood Dale, IL).

### Histological examination

Mice were deeply anesthetized with 64.8 mg/kg sodium pentobarbital (Kyoritsu Seiyaku Co., Tokyo, Japan) and perfused transcardially with potassium-free phosphate-buffered saline (PBS) followed by 4% paraformaldehyde. Bladders were removed, postfixed overnight in 4% paraformaldehyde, and embedded in paraffin (Sakura Finetek Japan, Tokyo, Japan). Paraffin-embedded tissues were cut into 5 μm sections and stained with hematoxylin and eosin (HE) using standard procedures. Histopathological examination was performed with a light microscope (BX-53F; OLYMPUS, Tokyo, Japan).

### Real-time RT-PCR

Anesthetized mice were perfused transcardially with PBS and bladders, the L5-S1 dorsal root ganglion (DRG), and the L5-S1 spinal cord were removed, flash-frozen in liquid nitrogen, and stored at −80°C until use. Total RNA was isolated from tissues with ISOGEN reagent (Nippon Gene, Tokyo, Japan), and cDNAs were synthesized with a ReverTra Ace qPCR RT Kit (Toyobo, Osaka, Japan). Real-time quantitative PCR was performed using the StepOne real-time PCR system (Life Technologies, Carlsbad, CA) with 20 μL reactions containing 1 μg of total RNA and the THUNDERBIRD SYBR qPCR Mix (Toyobo). Oligonucleotide primer pairs for 18S rRNA (5′-GCAATTATTCCCCATGAACG-3′ and 5′-GGCCTCACTAAACCATCCAA-3′), *Trpa1* (5′-TGAGATCGACCGGAGT-3′ and 3′-TGCTGAAGGCATCTTG-5′), *c-fos* (5′-CCGAAGGGAACGGAAT-3′ and 3′-TGCAACGCAGACTTCT-5′), glutathione peroxidase 1 (GPx1; 5′-GTTTCCCGTGCAATCAGTTC-3′ and 3′-CAGGTCGGACGTACTTGAGG-5′), and catalase (5′-GCGGATTCCTGAGAGAGTGG-3′ and 3′-TGTGGAGAATCGAACGGCAA-5′) were used. The results for each gene were normalized relative to 18S rRNA levels measured in parallel in each sample.

### Statistical analysis

Data are expressed as means ± S.E.M. Statistical analysis was performed with the GraphPad Prism 6 program (GraphPad Software, La Jolla, CA). Unpaired *t*-tests or Mann-Whitney *U*-tests were used to determine mRNA expression levels. The number of voids, freezing time, and distance moved were analyzed with two-way ANOVA, followed by the Tukey *post-hoc* test. In all cases, statistical significance was defined by a *p*-value < 0.05.

## Results

### Effect of TRPA1 deletion on the number of voids in H_2_O_2_-induced cystitis mice

**Figure 1:**
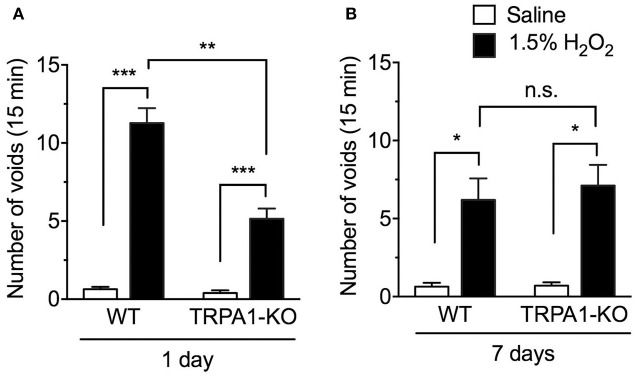
Number of voids in H_2_O_2_-injected wild-type (WT) and TRPA1-KO mice. WT or TRPA1-KO mice were injected intravesically with saline or 1.5% H_2_O_2_. At 1 day **(A)** and 7 days **(B)** after injection, the number of voids was counted in a 15 min period. Values are means ± S.E.M. for each group of 10–27 mice. ^*^p < 0.05, ^**^p < 0.01, ^***^p < 0.001 (n.s., not significant).

The number of voids was measured in WT and TRPA1-KO mice at 1 and 7 days after intravesical injection of saline (controls) or H_2_O_2_. Consistent with our previous report (Homan et al., 2013), an intravesical injection of 1.5% H_2_O_2_ significantly increased the number of voids 1 day after injection [*F*_(1, 69)_ = 71.82, *p* < 0.001]. This increase was significantly suppressed in TRPA1-KO mice [*F*_(1, 69)_ = 12.30, *p* < 0.001]. Both H_2_O_2_-injected WT and TRPA1-KO groups exhibited a significant increase in the number of voids compared with saline-injected WT and TRPA1-KO groups, respectively, and the number of voids in H_2_O_2_-injected TRPA1-KO mice was significantly lower than in H_2_O_2_-injected WT mice (Figure 1A). At 7 days after injection, the number of voids was significantly increased in H_2_O_2_-injected groups [*F*_(1, 67)_ = 16.31, *p* < 0.001], with significant increases observed in both WT and TRPA1-KO mice. However, there was no significant difference between WT and TRPA1-KO H_2_O_2_-injected groups [*F*_(1, 67)_ = 0.1099, *p* = 0.7413; Figure 1B].

### Effect of TRPA1 deletion on visceral pain-related behaviors in H_2_O_2_-induced cystitis mice

**Figure 2:**
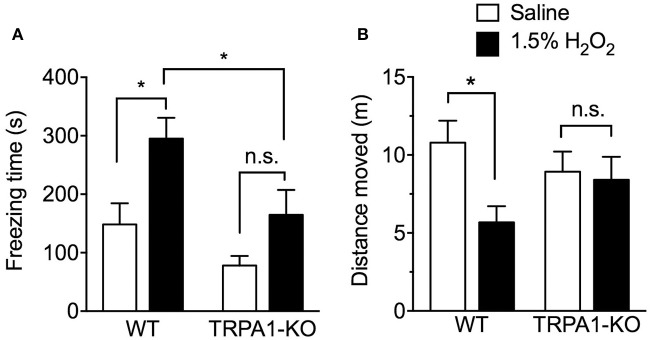
Spontaneous locomotor activities in H_2_O_2_-injected WT and TRPA1-KO mice. WT or TRPA1-KO mice were injected intravesically with saline or 1.5% H_2_O_2_. At 3 h after injection, freezing time **(A)** and distance moved **(B)** were measured in a 15 min period. Values are means ± S.E.M. for each group of 10–13 mice. ^*^p < 0.05 (n.s., not significant).

Reduced spontaneous locomotor activity in rodents is considered evidence of visceral pain-related behavior, as previously reported in a cyclophosphamide-induced cystitis mouse model (Miki et al., 2011). We previously reported a decrease in spontaneous locomotor behavior at only 3 h after intravesical H_2_O_2_ injection (Dogishi et al., 2015). In the present study, to examine the effect of TRPA1-KO on visceral pain-related behavior, spontaneous locomotor activities including freezing time and distance moved were analyzed over a 15 min period in freely moving WT and TRPA1-KO mice at 3 h after H_2_O_2_ injection. Intravesical H_2_O_2_ injection significantly increased freezing time [*F*_(1, 39)_ = 8.323, *p* < 0.01] and reduced the distance moved [*F*_(1, 39)_ = 4.717, *p* < 0.05] in WT but not in TRPA1-KO mice. TRPA1 deficiency significantly reduced freezing time [*F*_(1, 39)_ = 11.23, *p* < 0.01]. The freezing time in WT mice was significantly increased in the H_2_O_2_-injected group compared with the saline-injected control group, but a significant increase was not observed in H_2_O_2_-injected TRPA1-KO mice. Furthermore, the freezing time in H_2_O_2_-injected TRPA1-KO mice was significantly shorter than that in H_2_O_2_-injected WT mice (Figure 2A).

Similarly, H_2_O_2_-injected WT mice displayed a significant decrease in the distance moved compared with saline-injected WT mice. In TRPA1-KO mice, no significant difference was observed between saline- and H_2_O_2_-injected groups, and moving distance in the H_2_O_2_-injected group was increased compared with H_2_O_2_-injected WT mice, but not significantly (Figure 2B).

### Effect of TRPA1 deletion on upregulation of *c-fos* mRNA in the spinal cord of H_2_O_2_-induced cystitis mice

**Figure 3:**
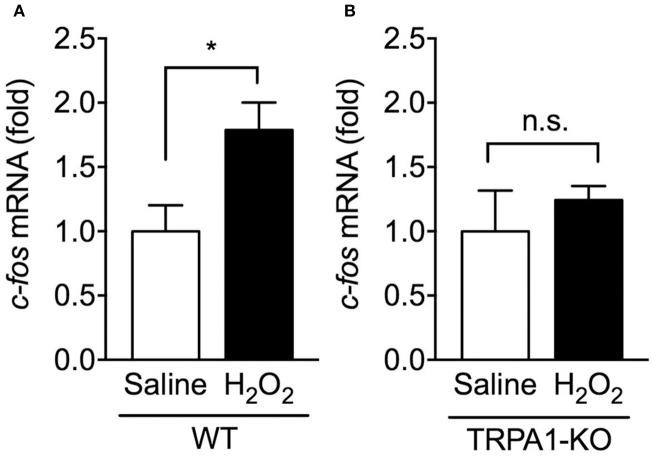
Expression levels of *c-fos* mRNA in the spinal cord of H_2_O_2_-injected WT and TRPA1-KO mice. WT **(A)** or TRPA1-KO **(B)** mice were injected intravesically with saline or 1.5% H_2_O_2_. At 1 day after injection, the L5-S1 spinal cord was removed, and *c-fos* mRNA levels were measured by real-time RT-PCR. The values were normalized against 18S rRNA mRNA levels and presented relative to those of saline-injected mice (set as 1). Values are means ± S.E.M. for each group of 5–14 mice. ^*^p < 0.05 (n.s., not significant).

Activation of bladder sensory neurons responsible for bladder hyperactivity and pain-related behaviors is correlated with the induction of *c-fos* mRNA expression, an immediate early gene, in the spinal cord (Avelino et al., 1999; Dinis et al., 2004). To determine whether TRPA1 deletion affects neuronal activity in the spinal cord caused by H_2_O_2_-induced cystitis, *c-fos* mRNA levels in the L5-S1 spinal cord, the area of termination of most bladder afferents (Nadelhaft and Booth, 1984), were examined 1 day after intravesical saline or H_2_O_2_ injection. In WT mice, H_2_O_2_ injection caused a significant upregulation in the relative expression of *c-fos* mRNA compared with the saline-injected control group. By contrast, in TRPA1-KO mice, there was no significant difference between saline- and H_2_O_2_-injected groups (Figure 3).

### Effect of TRPA1 deletion on histopathological changes in the bladder of H_2_O_2_-induced cystitis mice

**Figure 4:**
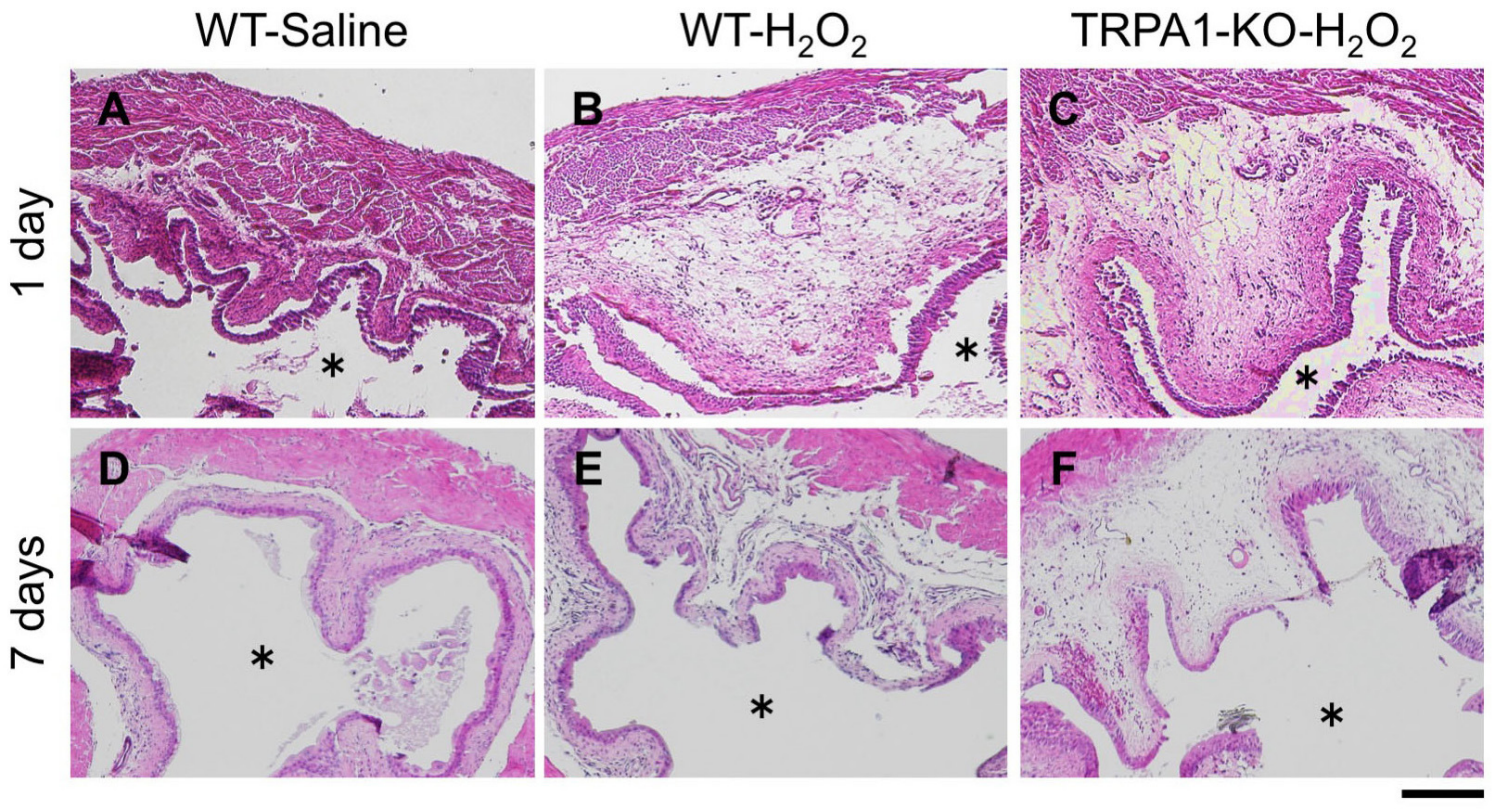
Histopathological examination of the bladders of H_2_O_2_-injected WT and TRPA1-KO mice. WT **(A,B,D,E)** or TRPA1-KO **(C,F)** mice were injected intravesically with saline **(A,D)** or 1.5% H_2_O_2_ **(B,C,E,F)**. At 1 day **(A–C)** and 7 days **(D–F)** after injection, bladders were removed and fixed, and tissue sections (5 μm) were stained with hematoxylin and eosin. Asterisks indicate the bladder lumen. Scale bar = 200 μm.

Cystitis induced by intravesical H_2_O_2_ injection was histopathologically examined by HE staining of the bladder of WT and TRPA1-KO mice. In H_2_O_2_-injected mice, severe edematous thickening in the submucosa was observed compared with the saline-injected control group at 1 day after injection, which was partially alleviated by 7 days after injection in both WT and TRPA1-KO mice. There was no difference in histopathological changes between WT and TRPA1-KO mice (Figure 4).

### Expression of *Trpa1* mRNA in the bladder and DRG of H_2_O_2_-induced cystitis mice

**Figure 5:**
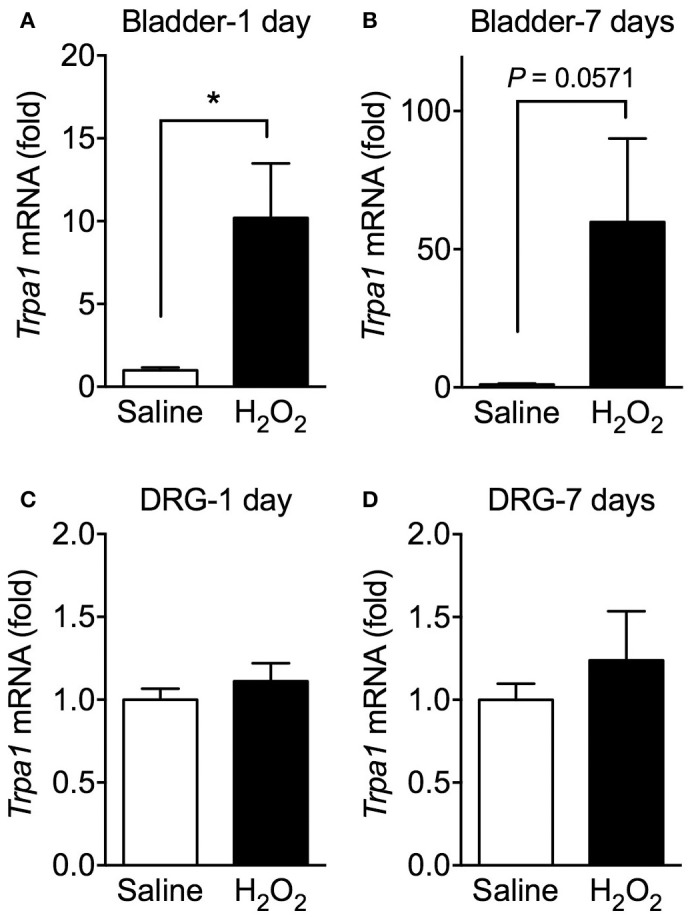
Expression levels of *Trpa1* mRNA in the urinary bladder and DRG of H_2_O_2_-injected mice. WT mice were injected intravesically with saline or 1.5% H_2_O_2_. At 1 day **(A,C)** or 7 days **(B,D)** after injection, bladders and the L5-S1 bilateral DRG were removed and *Trpa1* mRNA levels in the bladder **(A,B)** and DRG **(C,D)** were measured by real-time RT-PCR. The values were normalized against 18S rRNA mRNA levels and presented relative to those of the saline-injected group (set as 1). Values are means ± S.E.M. for each group of 3–6 mice. ^*^p < 0.05.

The effects of H_2_O_2_ injection on *Trpa1* mRNA levels in the urinary bladder and L5-S1 DRG were examined. Intravesical H_2_O_2_ injection drastically elevated the relative expression levels of *Trpa1* mRNA in the bladder on day 1 and 7. By contrast, there were no differences in the expression levels of *Trpa1* mRNA in the L5-S1 DRG between saline- and H_2_O_2_-injected groups at 1 and 7 days after injection (Figure 5).

## Discussion

In the present study, using an intravesical H_2_O_2_-induced long-lasting cystitis mouse model (Homan et al., 2013), we showed that TRPA1 is involved in initial bladder hyperactivity, but apparently not in the pathological processes involved in long-lasting cystitis, since (1) TRPA1 deletion reduced the initial increase in the number of voids and the decrease in spontaneous locomotor behaviors, which were accompanied by a reduction in *c-fos* mRNA upregulation in the spinal cord; (2) TRPA1 deletion had no effect on the delayed frequent urination; (3) TRPA1 deletion had no effect on histopathological changes in the urinary bladder at 1 or 7 days after injection. Furthermore, we found that *Trpa1* mRNA levels in the urinary bladder were drastically increased at 1 and 7 days after H_2_O_2_ injection, but levels were not altered in the DRG.

We confirmed that an intravesical injection of H_2_O_2_ produced long-lasting frequent urination, visceral pain-related behaviors, and bladder inflammation, as previously reported (Homan et al., 2013; Dogishi et al., 2015). Since H_2_O_2_ can activate TRPA1 (Andersson et al., 2008; Sawada et al., 2008), it is conceivable that H_2_O_2_ injected intravesically could directly stimulate TRPA1 in the bladder, leading to the generation of long-lasting cystitis. However, the present findings showed no apparent differences in histopathological changes in the urinary bladder between WT and TRPA1-KO mice injected with H_2_O_2_. Thus, direct stimulation of bladder TRPA1 by exogenous H_2_O_2_ appears not to play a major role in the induction of cystitis. Since the H_2_O_2_ solution was immediately drained from the bladder at 30 min after injection, and because H_2_O_2_ remaining in the bladder was rapidly degraded, H_2_O_2_-induced cystitis appears to be caused by non-selective insults to the bladder wall, probably by lipid peroxidation, protein oxidation, and DNA damage, as we discussed previously (Homan et al., 2013). In addition, we confirmed that TRPA1 deletion had no effects on the mRNA expression levels of antioxidant enzymes, GPx1, and catalase (Supplementary Figure 1).

The present behavioral experiments revealed that initial bladder hyperactivity, including frequent urination and visceral pain-related behaviors, was mediated, at least in part, through TRPA1 activation. In the lower urinary tract, sensations in the urinary bladder are conveyed to the spinal cord through primary sensory afferent neurons consisting of two types of fibers; myelinated (Aδ) and unmyelinated (C). It is well-known that C-fibers respond to noxious stimuli, while Aδ-fibers respond to bladder filling under physiological conditions (Fowler et al., 2008). Several pieces of evidence suggest that intravesical resiniferatoxin- or capsaicin-induced desensitization of C-fibers results in an increased bladder capacity and reduced bladder pain perception through inactivation of spinal cord neurons in an animal model of acute cystitis (Dinis et al., 2004; Saitoh et al., 2009). Taken together with the present results showing the loss of *c-fos* mRNA upregulation in the spinal cord of H_2_O_2_-injected TRPA1-KO mice, this suggests that activation of C-fibers through TRPA1 stimulation enhances the activity of spinal cord neurons, resulting in frequent urination and abdominal visceral pain during the initial phase of long-lasting cystitis. Acute damage to bladder urothelial cells induced by exogenous H_2_O_2_ injection causes hyperpermeability of the urothelial barrier (Homan et al., 2013). Thus, the submucosa is exposed to irritants in the urine, which may activate TRPA1 expression on the bladder terminal of C-fibers. Alternatively, H_2_O_2_-induced acute inflammation of the bladder may be accompanied by bladder vascular hyperpermeability and infiltration of abundant inflammatory cells, including neutrophils, into the submucosa (Homan et al., 2013; Dogishi et al., 2017). Several lines of evidence suggest that ROS produced from infiltrated inflammatory cells contribute to bladder hyperactivity (Chien et al., 2003; Masuda et al., 2008). Consequently, it is conceivable that excessive ROS produced from infiltrated inflammatory cells in the submucosa may activate TRPA1. Under severe initial bladder inflammation, it is possible that TRPA1 may be sensitized to ROS by various inflammatory mediators (Gouin et al., 2017).

By contrast, TRPA1 appears not to play a major role during the latter stages of long-lasting cystitis. At 7 days after intravesical H_2_O_2_ injection, frequent urination was partially alleviated, although it still persisted, and the decrease in spontaneous locomotor behaviors ceased, as reported previously (Homan et al., 2013; Dogishi et al., 2015). The observed severe edematous thickening of the submucosa was partially alleviated by 7 days after injection. Furthermore, we previously reported that the urothelial damage and hyperpermeability are recovered within several days, while bladder inflammation, such as accumulation of inflammatory cells and increased expression of inflammatory cytokines, persisted (Homan et al., 2013). Under such long-lasting inflammatory bladder conditions, excessive ROS production and/or sensitization of TRPA1 to ROS in the bladder may be recovered. We previously reported that bladder tissue remodeling, such as hyperplasia of the urothelium, vascularization, and fibrosis, is induced in the late phase of long-lasting cystitis (Homan et al., 2013; Dogishi et al., 2017). In addition to hyperactivity of bladder sensory neurons induced by long-lasting inflammation, bladder structural changes may affect the micturition function, leading to frequent urination. However, it is difficult to perform cystometric analysis in the present mouse cystitis model, although we could measure intercontraction interval and intravesical pressure in intravesical H_2_O_2_-induced rat cystitis model (Dogishi et al., 2017). Such technical problems by using genetically-modified mice limit to analyze the urodynamics in the mouse cystitis model. Further detailed investigations including cystometry will be needed to elucidate the roles of TRPA1 in the long-lasting bladder hyperactivity.

Recent evidence suggests that activation of TRPA1 may cause and/or enhance neurogenic inflammation (Gouin et al., 2017). However, the present results suggest that TRPA1 is not responsible for the occurrence and maintenance of bladder inflammation. Consistently, a TRPA1 antagonist attenuates visceral nociception in an ifosfamide-induced cystitis model, although ifosfamide-induced bladder inflammation is not suppressed (Pereira et al., 2013).

In the lower urinary tract, TRPA1 is expressed in both C-fibers and the bladder epithelium (Streng et al., 2008; Wu et al., 2010). This raises the question of which sites expressing TRPA1 are associated with initial bladder hyperactivity. In the present study, we found that *Trpa1* mRNA levels were drastically upregulated in the urinary bladder from the initial to the late phases, but not in the L5-S1 DRG. Consistent with these findings, upregulation of *Trpa1* mRNA was reported in the urinary bladder of patients with bladder outlet obstruction or IC/BPS (Du et al., 2008; Homma et al., 2013), suggesting that upregulation of TRPA1 expression in the urinary bladder may be pathologically correlated with bladder disorders. Given these expression changes, it is possible that TRPA1 expressed in the urinary bladder, rather than in the DRG, may be responsible for initial bladder hyperactivity. However, this interpretation may be a hasty judgement because the involvement of TRPA1 was observed only during the initial phase, but not in the late phase, although upregulation of *Trpa1* mRNA persisted until at least 7 days after injection. Under inflammatory conditions, the sensitivity of TRPA1 in the DRG is reportedly enhanced without changes in expression levels, and this allegedly contributes to hyperalgesia (Zhou et al., 2013). Thus, functional sensitization of TRPA1 expressed in the DRG may contribute to initial bladder hyperactivity, including frequent urination and visceral pain-related behaviors. Further investigation is therefore required to identify the sites of TRPA1 expression responsible for the pathology of long-lasting cystitis.

In conclusion, the present study revealed that TRPA1 contributes to initial bladder hyperactivity, affecting the frequency of urination and abdominal visceral pain, but it does not appear to play a major role in the pathology of long-lasting cystitis. Therapeutic strategies targeting TRPA1 may be effective for minimizing bladder hyperactivity in acute cystitis, but its usefulness for chronic cystitis may be limited.

## Author contributions

SO, TN, and SK designed the project. SO, KD, MKo and MKa performed the experiments. SO, KD, MKo, HS, KN, and TN analyzed the data. SO and TN wrote the manuscript. SK supervised the experiments and finalized the manuscript.

### Conflict of interest statement

The authors declare that the research was conducted in the absence of any commercial or financial relationships that could be construed as a potential conflict of interest.
