# Supplementary material for: Pathophysiological Role of Transient Receptor Potential Ankyrin 1 in a Mouse Long-Lasting Cystitis Model Induced by an Intravesical Injection of Hydrogen Peroxide
Source: Front Physiol. 2017 Nov 7;8:877. doi: 10.3389/fphys.2017.00877 (PMC5715365; doi:10.3389/fphys.2017.00877)
Supplement: Supplementary file 1 [file Image1.PDF]

Supplementary Material

Pathophysiological role of transient receptor potential ankyrin 1 in a mouse long-lasting cystitis model induced by an intravesical injection of hydrogen peroxide

Shohei Oyama, Koji Dogishi, Mizuki Kodera, Masashi Kakae, Kazuki Nagayasu, Hisashi Shirakawa, Takayuki Nakagawa, Shuji Kaneko

*** Correspondence:** Takayuki Nakagawa: tknakaga@kuhp.kyoto-u.ac.jp

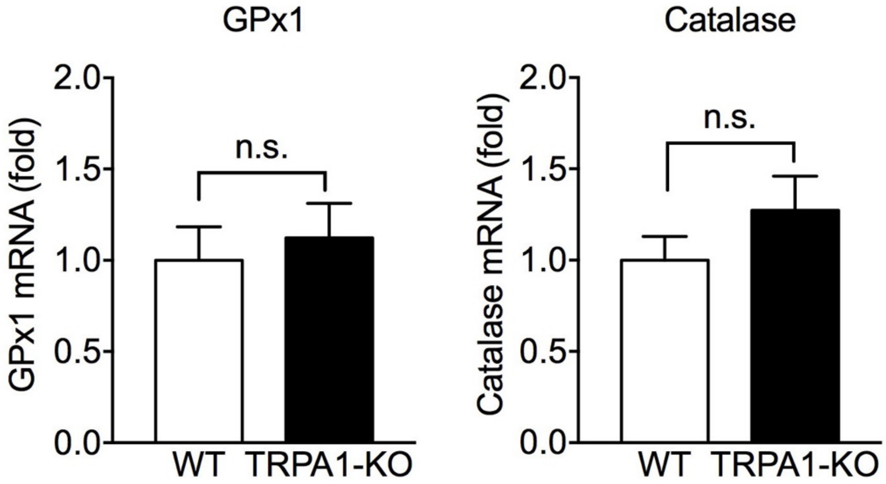

Supplementary Figure 1. No effect of TRPA1 deletion on the mRNA expression levels of glutathione peroxidase 1 (GPx1) and catalase in the bladder. Bladder was removed from WT or TRPA1-KO mice, and the levels of GPx1 and catalase mRNAs were measured by real-time RT-PCR. The values were normalized against 18S rRNA mRNA levels and presented relative to those of WT mice (set as 1). Values are means ± S.E.M. for each group of 6 mice. n.s. = not significant (Student's *t*-test).
